# Prognostic value of peak work rate indexed by left ventricular diameter

**DOI:** 10.1038/s41598-023-35976-x

**Published:** 2023-05-31

**Authors:** Charlotte Eklund Gustafsson, Magnus Ekström, Martin Ugander, Lars Brudin, Anna Carlén, Kristofer Hedman, Thomas Lindow

**Affiliations:** 1 Department of Clinical Physiology, Research and Development, Växjö Central Hospital, Region Kronoberg, Växjö, Sweden; 2 Department of Clinical Sciences Lund, Respiratory Medicine and Allergology, Faculty of Medicine, Lund University, Lund, Sweden; 3 Kolling Institute, Royal North Shore Hospital, University of Sydney, Sydney, Australia; 4 Department of Clinical Physiology, Karolinska University Hospital, Karolinska Institute, Stockholm, Sweden; 5 Department of Clinical Physiology, Kalmar County Hospital, Kalmar, Sweden; 6 Department of Clinical Physiology in Linköping, Linköping University, Linköping, Sweden; 7 Department of Health, Medicine and Caring Sciences, Linköping University, Linköping, Sweden; 8 Clinical Physiology, Clinical Sciences, Lund University, 221 00 Lund, Sweden

**Keywords:** Echocardiography, Cardiovascular diseases, Heart failure

## Abstract

Left ventricular diameter (LVEDD) increases with systematic endurance training but also in various cardiac diseases. High exercise capacity associates with favorable outcomes. We hypothesized that peak work rate (W_peak_) indexed to LVEDD would carry prognostic information and aimed to evaluate the association between W_peak_/LVEDD_rest_ and cardiovascular mortality. W_peak_/LVEDD_rest_ (W/mm) was calculated in patients with an echocardiographic examination within 3 months of a maximal cycle ergometer exercise test. Low W_peak_/LVEDD_rest_ was defined as a value below the sex- and age-specific 5th percentile among lower-risk subjects. The association with cardiovascular mortality was evaluated using Cox regression. In total, 3083 patients were included (8.0 [5.4–11.1] years of follow-up, 249 (8%) cardiovascular deaths). W_peak_/LVEDD_rest_ (W/mm) was associated with cardiovascular mortality (adjusted hazard ratio (HR) 0.28 [0.22–0.36]), similar to W_peak_ in % of predicted, with identical prognostic strength when adjusted for age and sex (C-statistics 0.87 for both). A combination of low W_peak_/LVEDD_rest_ and low W_peak_ was associated with a particularly poor prognosis (adjusted HR 6.4 [4.0–10.3]). W_peak_/LVEDD_rest_ was associated with cardiovascular mortality but did not provide incremental prognostic value to W_peak_ alone. The combination of a low W_peak_/LVEDD_rest_ and low W_peak_ was associated with a particularly poor prognosis.

## Introduction

The left ventricle (LV) has adaptive properties in response to external demands, both in athletes and healthy individuals who develop increased LV dimensions as their exercise capacity and total heart volume increases^1–4^. However, LV dimensions may also increase due to cardiac disease (such as myocardial infarction, dilated cardiomyopathy and aortic or mitral regurgitation)^5^.

Peak oxygen consumption (peak VO2) measured during cardiopulmonary exercise testing (CPET) has been shown to be strongly related to cardiac dimensions^6–9^. In healthy individuals, larger cardiac dimensions are thus expected with higher peak VO_2_, or vice versa^10,11^. The absence of such a relation, i.e. increased cardiac dimensions but low peak VO_2_, is instead suggestive of cardiac disease, e.g. with left ventricular (LV) dilatation as an adaptation to pathological conditions^2^. Prior studies on the relation between cardiac dimensions and exercise capacity has been performed with CPET, i.e. with breathing gas analysis, cardiovascular magnetic resonance imaging/or echocardiography^2,6^. Given the strong relation between peak VO_2_ and peak work rate (W_peak_), cycle ergometer exercise testing could be used as a surrogate for CPET, offering a more widely available method for assessment of this relation^12^. No prior study has addressed the potential prognostic value of an abnormal relationship between W_peak_ and LV size.

Measures of exercise capacity are strongly associated with cardiovascular (CV) morbidity and mortality, both in patients with established CV disease and in those without^13–21^. We hypothesized that by combining the prognostic information of exercise capacity and LV dimensions, by indexing W_peak_ to LV end-diastolic diameter (LVEDD), a stronger association with CV mortality would emerge, compared to that for W_peak_ alone, in particular in patients who are affected by both a low W_peak_ and a large LVEDD. The aim of the current study was to evaluate if W_peak_/LVEDD_rest_ predicted CV mortality better than W_peak_ only.

## Methods

**Figure 1:**
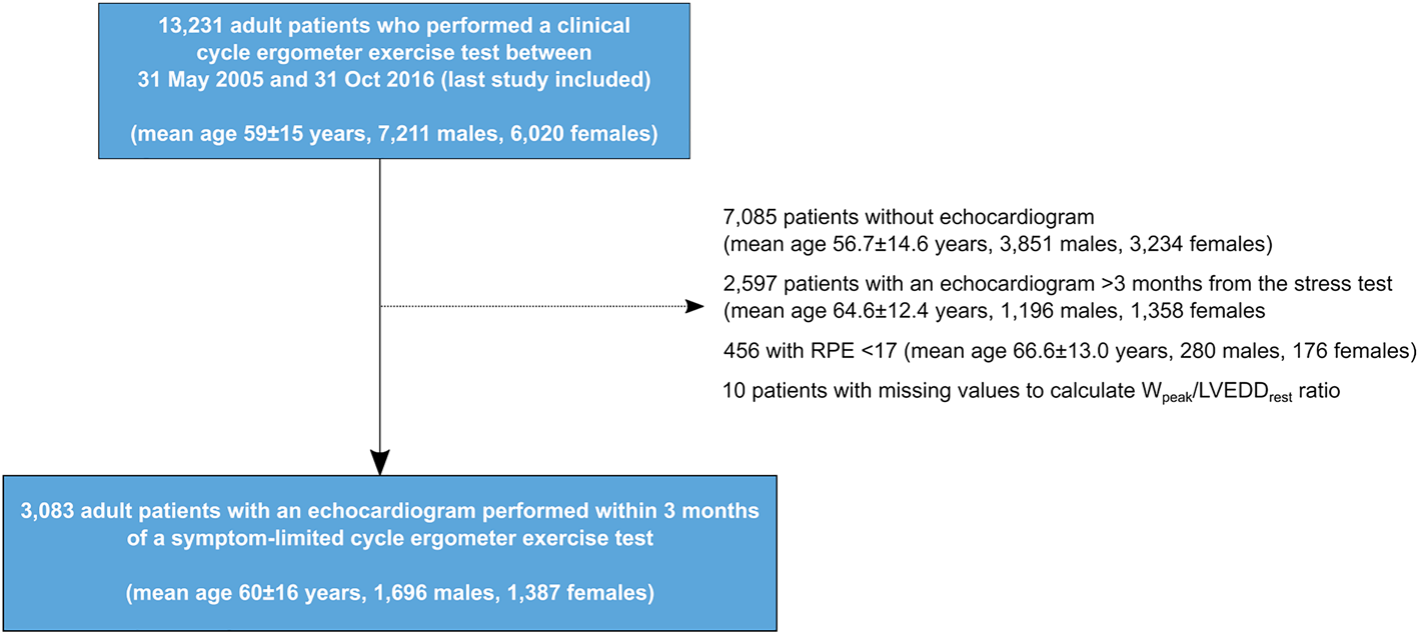
Flow chart of patient inclusion and exclusion in the study. RPE denotes rate of perceived exertion.

We performed a retrospective analysis of consecutive patients aged 18 years or older who were referred for a clinical cycle ergometer exercise test at the department of Clinical Physiology at Kalmar County Hospital, Sweden between 31 May 2005 and 31 Oct 2016. The exercise stress test database has been described in detail elsewhere, and forms the basis for the Swedish national recommendations for grading of exercise capacity during standardized exercise stress testing^13,22–25^. Within this database, patients who had performed an echocardiographic examination within 3 months from the date of the exercise stress test were included. Patients who did not reach a peak rating of perceived exertion (RPE) of 17/20, and patients with missing data on W_peak_ or LVEDD_rest_ were excluded. A flowchart of patient inclusion and exclusion is presented in Fig. 1.

To obtain survival status for each subject until study census date, the database was cross-linked with the mandatory Swedish Causes of Death Register (until 31 Dec 2019). Data on comorbidities, medications and hospital admission data were obtained through cross-linkage with the mandatory Swedish National Patient Register (until 31 Dec 2019)^26^. Definitions and diagnosis codes (International Classification of Diseases-10 (ICD-10)) are presented in Supplements (Table A). CV death was defined as death with an underlying cause within the ICD-10 chapter of CV disease (IX).

### Exercise testing

Exercise testing was performed using a standardized protocol on an electrically braked computer-controlled, regularly calibrated cycle ergometer (Rodby Inc, Karlskoga, Sweden). A 12-lead electrocardiogram was recorded during rest, exercise and recovery. Systolic blood pressure was measured in the supine position before and after exercise, seated on the bicycle before exercise, and every 2 min during exercise. RPE was reported every 2 min during exercise. Work rate was started at 40–100 W (men) or 30–50W (women), depending on the expected exercise capacity, with an incremental increase of 10–20 W/min^27^. Patients were encouraged to exercise until exhaustion. The test was terminated at the patient’s will or if predefined termination criteria were fulfilled (severe chest pain, ST depression ≥ 0.4 mV, decreasing systolic blood pressure or any malignant dysrhythmia). In order to take the effect of different work load increments on the achieved absolute W_peak_, into account, the achieved W_peak_ was re-calculated*,* if necessary*,* to a standard protocol with an increment of 15 W/min (men) and 10 W/min (women) by the following formula^28^: females: W_peak_ × (incremental workload used/10)^1/6^); males: W_peak_ × (incremental workload used/15)^1/6^. The re-calculated exercise capacity was then related to the Swedish reference material for standardized exercise stress testing (% of predicted W_peak_, which is adjusted for age, gender and height)^25^. ST-segment amplitudes were measured 60 ms following the J-point (ST60). Significant ST depression was defined as horizontal or down-sloping ST depression ≥ 0.1 mV in V5 during exercise or during the recovery phase. Heart rate recovery was defined as the difference in heart rate between the maximal heart rate and the heart rate 2 min after cessation of exercise^15,29^. If a patient had performed more than one test, only the most recent test was included.

### Echocardiography

Two-dimensional transthoracic resting echocardiography was performed using commercially available echocardiographic equipment. At end-diastole, LVEDD_rest_ (mm), septal and posterior wall thickness (IVS and PWT, mm) were obtained either from M-mode or 2D images in the parasternal long axis view. LV mass was calculated according to the Cube formula (LV mass (g) = 0.8 × 1.04 ([IVS + LVEDD + PWT]^3^ − LVEDD^3^) + 0.6) and presented after adjustment to body-surface (BSA)^30^. Left ventricular ejection fraction (LVEF) was reported either based on the Simpson biplane method, from M-mode data (Teichholtz formula), or by visual estimation^31^.

Aortic, mitral or tricuspid regurgitation were graded as none, mild, moderate or severe. Moderate aortic stenosis was defined as either an aortic valve (AV) maximal velocity by continuous Doppler ≥ 3.1–4.0 m/s or an AV mean gradient of 20–40 mm Hg, while severe aortic stenosis corresponded to an AV maximal velocity ≥ 4.0 m/s or an AV mean gradient ≥ 40 mm Hg. Early (E) and late (A) diastolic velocity over the mitral valve were measured using pulsed Doppler and the E/A ratio was calculated. Pulsed tissue Doppler imaging with a 2-mm sample volume placed in the myocardium at the septal and lateral mitral annulus (apical four-chamber view) was used to determine the average early diastolic myocardial velocity (e’), in order to calculate the E/e’ ratio. W_peak_/LVEDD_rest_ (W/mm) was calculated as the peak work rate at exercise stress testing divided by the end-diastolic LV diameter during resting echocardiography.

### Statistical analysis

Continuous variables were described using mean and standard deviation (SD). Comparisons of group means were performed using Student’s t test. Differences between groups were assessed using the χ^2^ test. The correlation between W_peak_ and LVEDD was analyzed using Pearson’s r and visualized using scatter plots, separately for all patients, lower-risk subjects, patients with moderate/severe left-sided valvular regurgitation, and for patients with heart failure.

The association between W_peak_/LVEDD_rest_ and CV mortality, and the association between W_peak_ in % of predicted, was analyzed using Cox proportional hazard regression models. Models were evaluated unadjusted; adjusted for age and sex; and adjusted for age, sex, peak systolic blood pressure, presence of ST depression, heart rate recovery, peak heart rate, LVEF, E/e’, heart failure, hypertension, previous myocardial infarction, diabetes mellitus, hyperlipidemia, and peripheral arterial disease. The choice of confounding variables was based on directed acyclic graphs and previous literature knowledge. The assumption of proportional hazards was confirmed using Schoenfeld’s residuals. Results are presented as hazard ratios (HR) with 95% confidence intervals (CI) and C statistics for the continuous W_peak_/LVEDD_rest_ and W_peak_.

We also present HR for combinations of low/normal (W_peak_/LVEDD_rest_ and low/normal W_peak_). Since no reference values for W_peak_/LVEDD_rest_ exist, an approximate age- and sex-specific lower limit of normal (LLN) for W_peak_/LVEDD_rest_ was defined among a subgroup of lower-risk subjects in this cohort, including only non-obese (BMI < 30 kg/m^2^) subjects with normal LVEF (≥ 55%), absence of moderate/severe valvular heart disease, without CV medications or known CV, renal, respiratory, or malignant disease. A low W_peak_ was defined as W_peak_ in % of predicted below the LLN according to the validated, Swedish reference material for exercise capacity^13,25^.

Natural cubic spline modelling was used to characterize the risk associated with W_peak_/LVEDD_rest_ and W_peak_ as a continuum, using four knots placed at the 5th, 25th, 75th and 95th percentiles.

Statistical significance was defined as a two-tailed *p*-value < 0.05. Statistical analysis was performed using R v. 3.5.3 (R Core Team (2021). R: A language and environment for statistical computing. R Foundation for Statistical Computing, Vienna, Austria (https://www.R-project.org/), example packages: Survival v. 3.1-12rms v. 6.3.0).

### Ethical approval

The study complies with the Declaration of Helsinki and was approved by the Swedish Ethical Review Authority (Dnr 2012/379-31; 2018/141-31 and 2020/00352). Informed consent was waived by the Swedish Ethical Review Authority.

### Permissions

The manuscript does not contain any reproduced material from other sources.

## Results

A total of 3083 patients (mean age 60 ± 16 years, 55% male) were included. During a median follow-up of 8.0 [IQR 5.4–11.1] years, 592 (19%) patients died of whom 249 (8%) died due to a CV cause. Baseline characteristics are presented in Table 1. An echocardiographic examination was performed within 0 [0–1] days from the exercise stress test.

**Table 1:** Baseline characteristics of the whole study population and grouped according to W_peak_/LVEDD_rest_ above or below the 5^th^ percentile of the lower-risk subgroup.

	All	W_peak_/LVEDD_rest_ > LLN*	W_peak_/LVEDD_rest_ < LLN	*p*
N (%)	3083	2012 (65.3)	1071 (34.7)	
Age, years	59.9 ± 16.0	55.2 ± 15.6	69.0 ± 12.6	< 0.001
Male sex, n (%)	1696 (55.0)	1075 (53.4)	621 (58.0)	0.017
Cycle ergometer exercise test variables				
Peak heart rate, bpm	146 ± 27	155 ± 23	129 ± 24	< 0.001
Peak systolic blood pressure, mmHg	190 ± 30	194 ± 28	181 ± 32	< 0.001
Heart rate at rest, bpm	73 ± 14	72 ± 14	74 ± 14	< 0.001
Heart rate recovery, bpm	30 ± 15	33 ± 15	23 ± 14	< 0.001
Peak work rate (W_peak_), W	151 ± 60	174 ± 57	108 ± 34	< 0.001
Peak work rate, % predicted	85 ± 19	94 ± 16	69 ± 13	< 0.001
Echocardiographic variables				
LVEDD, mm	48 ± 6	47 ± 5	49 ± 6	< 0.001
LVEDD indexed to BSA, mm/m^2^	25 ± 3	25 ± 3	26 ± 3	< 0.001
LA diameter, mm	40 ± 6	38 ± 6	42 ± 7	< 0.001
LA diameter indexed to BSA, mm/m^2^	21 ± 3	20 ± 3	22 ± 3	< 0.001
LVEF, %	63 ± 11	65 ± 11	59 ± 12	< 0.001
Low LVEF < 50%, n (%)	254 (8.3)	88 (4.4)	167 (15.6)	< 0.001
LVOT-VTI, cm	21 ± 4	21 ± 4	20 ± 5	< 0.001
Left ventricular mass, indexed to BSA, g/m^2^	99 ± 28	93 ± 23	111 ± 32	< 0.001
E/e' (cm/s)	11 ± 4	10 ± 4	12 ± 5	< 0.001
E/A ratio	1.2 ± 0.5	1.2 ± 0.5	1.0 ± 0.4	< 0.001
RV to RA pressure gradient, mmHg	23 ± 8	22 ± 6	26 ± 9	< 0.001
Aortic regurgitation (moderate/severe), n (%)	101 (3.3)	43 (2.1)	58 (5.4)	< 0.001
Mitral regurgitation (moderate/severe), n (%)	270 (8.8)	115 (5.7)	155 (15.0)	< 0.001
Aortic stenosis (moderate/severe), n (%)	103 (3.3)	50 (2.5)	53 (4.9)	< 0.001
Clinical HF classification				
HFpEF, n (%)	54 (1.8)	40 (2.0)	14 (1.3)	
HFmrEF, n (%)	132 (4.3)	43 (2.1)	90 (8.4)	
HFrEF, n (%)	122 (4.0)	45 (2.2)	77 (7.2)	
Comorbidities				
Hypertension, n (%)	293 (9.5)	206 (10.2)	87 (8.1)	0.07
Ischemic heart disease, n (%)	218 (7.1)	148 (7.4)	71 (6.6)	0.50
Previous acute myocardial infarction, n (%)	109 (3.6)	72 (3.6)	38 (3.5)	1.0
Malignancy, n (%)	207 (6.7)	130 (6.5)	78 (7.3)	0.43
Cerebrovascular disease, n (%)	37 (1,2)	25 (1.2)	12 (1.1)	1.0
Chronic obstructive pulmonary disease, n (%)	46 (1.5)	32 (1.2)	14 (1.3)	0.65
Medications				
ACE inhibitors, n (%)	585 (19.0)	294 (14.6)	292 (27.3)	< 0.001
Beta blockers, n (%)	994 (32.2)	498 (24.7)	496 (46.3)	< 0.001
Loop diuretics, n (%)	298 (9.7)	80 (4.0)	218 (20.4)	< 0.001
Anti-diabetics, n (%)	169 (5.5)	76 (3.8)	93 (8.7)	< 0.001
Insulin, n (%)	150 (4.9)	61 (3.0)	89 (8.3)	< 0.001
Calcium antagonists, n (%)	413 (13.4)	188 (9.3)	225 (21.0)	< 0.001
Thiazide diuretics, n (%)	194 (6.3)	96 (4.8)	98 (9.2)	< 0.001
Anti-thrombotic, n (%)	722 (23.4)	341 (16.9)	381 (35.6)	< 0.001
Nitrates, n (%)	367 (11.9)	169 (8.4)	198 (18.5)	< 0.001
Anti-coagulant, n (%)	237 (7.7)	85 (4.2)	152 (14.2)	< 0.001
Lipid-lowering, n (%)	657 (21.3)	318 (15.7)	339 (31.7)	< 0.001

Continuous variables are presented as mean ± standard deviation. Categorical variables are presented as number of patients (%).

*ACE* angiotensin-converting enzyme, *BSA* body surface area, *E/A* early/late diastolic velocity ratio over the mitral valve, *E/e'* early diastolic mitral velocity /early diastolic myocardial velocity *e’* HF: heart failure; *HFmrEF* heart failure with mid-range ejection fraction, *HFpEF* heart failure with preserved ejection fraction, *HFrEF* heart failure with reduced ejection fraction, *LA* left atrial, *LVEDD* left ventricular end-diastolic dimension, *LVEF* left ventricular ejection fraction, *LVOT-VTI* left ventricular outflow tract velocity time integral.

*LLN: sex- and age-specific 5th percentile for W_peak_/LVEDD_rest_ in lower-risk subjects (Table 2).

The mean W_peak_/LVEDD_rest_ was 3.1 ± 1.2 W/mm in the whole study group. Males had higher W_peak_/LVEDD_rest_ compared to females (3.6 ± 1.2 W/mm vs. 2.6 ± 0.8 W/mm, *p* < 0.001). W_peak_/LVEDD_rest_ was lower in older age groups among both sexes (Supplemental Fig. A).

Mean W_peak_/LVEDD_rest_ in HF patients was 2.6 ± 1.1 W/mm, 2.6 ± 0.9 W/mm in patients with at least moderate mitral or aortic regurgitation, and 3.3 ± 1.1 W/mm in patients with neither HF nor mitral or aortic regurgitation (*p* < 0.001 for both comparisons).

**Figure 2:**
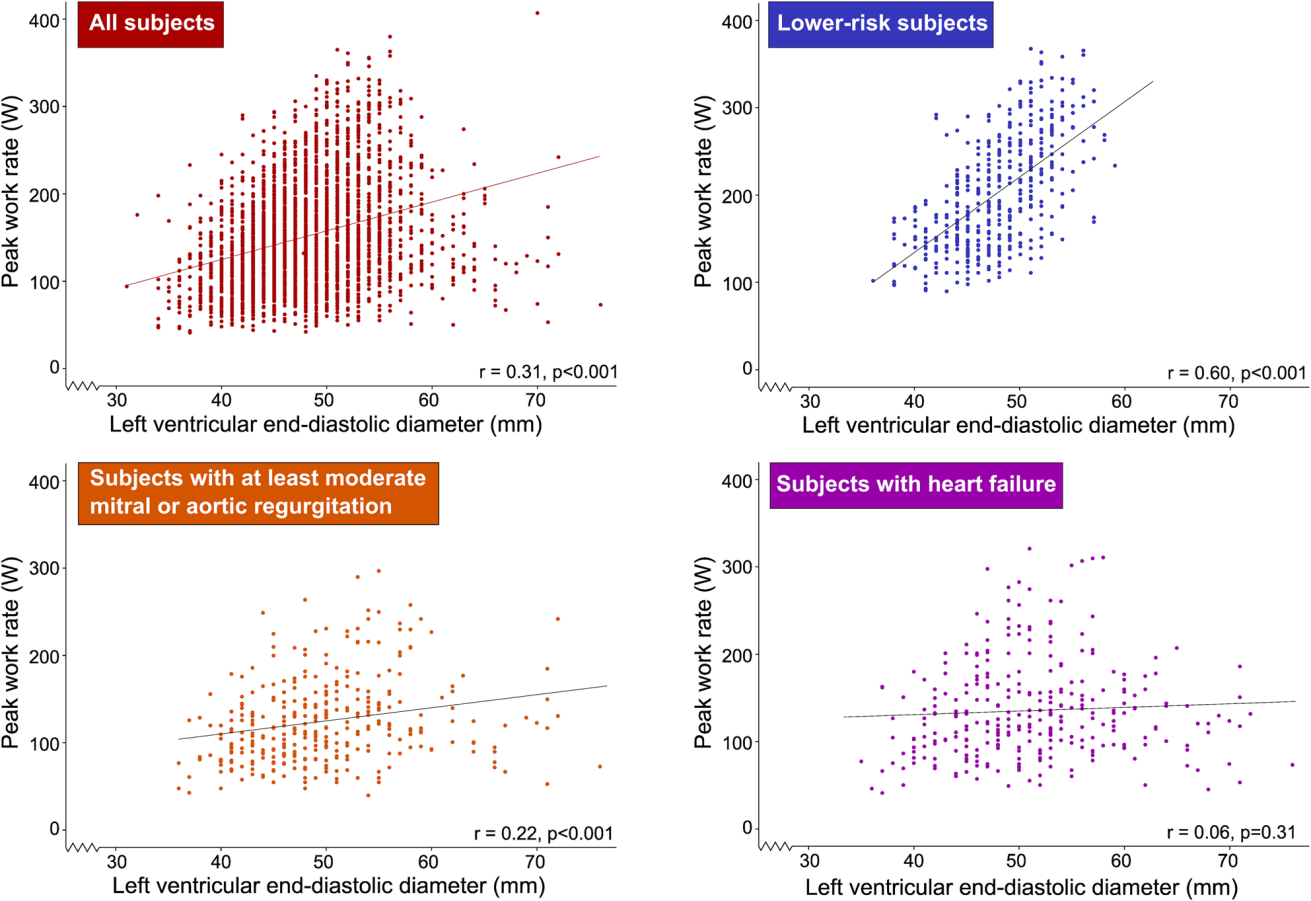
The relation between peak work rate and left ventricular size in all subjects (n = 3083) (upper left panel, red), in low-risk patients (n = 460) (upper right panel, blue), patients with at least moderate mitral or aortic regurgitation (n = 346) (lower left panel, orange), and in heart failure patients (n = 309) (lower right panel, purple).

A total of 460 patients (15%) met the definition as lower-risk subjects. Among these, W_peak_ was moderately correlated with LVEDD_rest_ (r = 0.61, *p* < 0.001) (Fig. 2), while there was no clinically significant correlation between W_peak_ and LVEDD_rest_ among patients with HF or valvular heart disease (r = 0.06, *p* = 0.31 and r = 0.22, *p* < 0.001 respectively). A low W_peak_/LVEDD_rest_, defined as less than age- and sex specific 5th percentile of the lower-risk subgroup (Table 2), was found in 1,362 patients (44.2%). Compared to subjects with W_peak_/LVEDD_rest_ above this threshold, patients with a low W_peak_/LVEDD_rest_ had a higher prevalence of low LVEF < 50% (14.2% vs. 3.5%, *p* < 0.001), while they had greater E/e’ and LV mass (12 vs. 9, *p* < 0.001; 109 g/m^2^ vs. 92 g/m^2^, *p* < 0.001 respectively), Table 1.

**Table 2:** Distribution of W_peak_/LVEDD_rest_ (W/mm) among lower risk subjects presented in percentiles.

	Percentiles		
	5th	50th	95th
Males			
< 40 years	4.22	5.24	6.41
40–60 years	3.64	4.84	6.35
> 60 years	2.99	3.99	5.33
Females			
< 40 years	2.90	3.56	4.51
40–60 years	2.46	3.21	4.44
> 60 years	2.15	2.78	3.59

Higher W_peak_/LVEDD_rest_ was associated with reduced CV mortality (unadjusted HR: 0.26 [0.22–0.31] per 1 W/mm), Table 3, Fig. 4. C-statistics were numerically higher for W_peak_/LVEDD_rest_ than for W_peak_ in % of predicted (0.80 [0.78–0.82] vs. 0.78 [0.75–0.81], *p* = 0.15). C-statistics were identical after adjusting for age and sex (C-statistics 0.87 [0.85–0.89] in both). Indexing W_peak_ to LVEDD/body surface area did not improve its performance (C-statistic 0.76 [0.73–0.79]).

**Table 3:** Associations between peak work rate to left-ventricular end-diastolic diameter at rest (W_peak_/LVEDD_rest_) and W_peak_ (% of predicted) and LVEDD_rest_, respectively, and cardiovascular mortality (n = 3083 (249 events)).

	Unadjusted	Adjusted for age and sex	Final model*
	HR [95%CI]	HR [95%CI]	HR [95%CI]
	C statistic [95%CI]	C statistic [95%CI]	C statistic [95%CI]
W_peak_/LVEDD_rest_ (W/mm)	0.26 [0.22–0.31]	0.22 [0.18–0.28]	0.29 [0.22–0.37]
	0.80 [0.78–0.82]	0.87 [0.85–0.89]	0.88 [0.86–0.90]
W_peak_ (% of predicted)	0.94 [0.94–0.95]	0.95 [0.94–0.96]	0.96 [0.95–0.97]
	0.78 [0.75–0.81]	0.87 [0.85–0.89]	0.88 [0.86–0.90]
LVEDD_rest_ (mm)	1.06 [1.04–1.08]	1.08 [1.06–1.10]	1.04 [1.02–1.06]
	0.58 [0.53–0.62]	0.82 [0.77–0.85]	0.86 [0.84–0.89]

*HR* hazard ratio, *LVEDD* left ventricular end-diastolic diameter, *W* Watt, *W*_peak_ peak work rate.

*Adjusted for age, sex, peak systolic blood pressure, ST depression, heart rate recovery, peak heart rate, left ventricular ejection fraction, E/e, heart failure, hypertension, myocardial infarction, diabetes mellitus, hyperlipidemia, and peripheral arterial disease.

**Figure 3:**
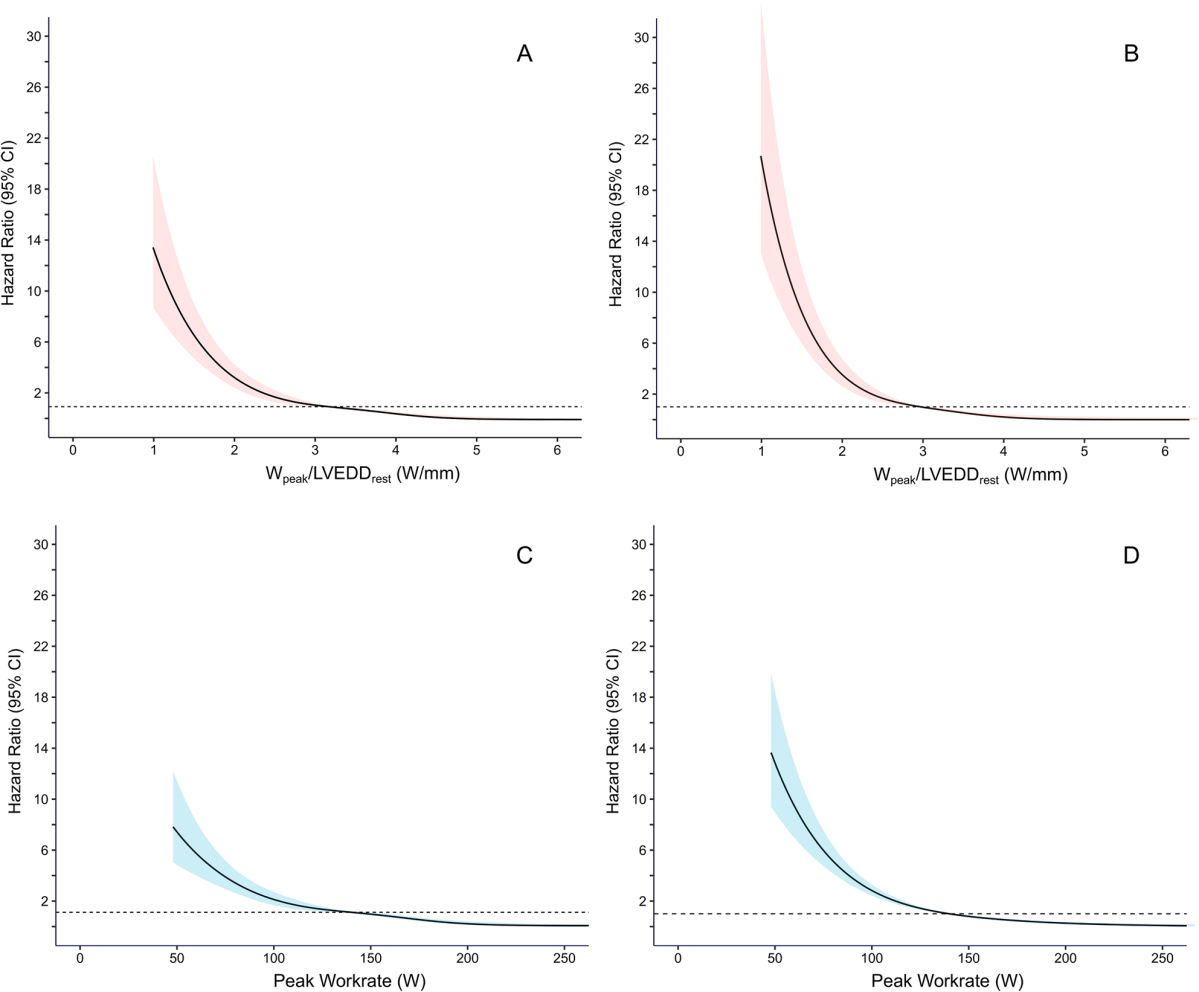
Impact of decreasing W_peak_/LVEDD_rest_ and W_peak_ alone on the risk of cardiovascular death. The hazard ratios (95% confidence intervals) were calculated using Cox regression and modelled with natural cubic splines with four knots (percentiles: 5th, 25th, 75th, 95th) and presented as unadjusted estimates (left panels) and adjusted for age and sex (right panels). W_peak_/LVEDD_rest_ showed a substantial risk increase with low values both for adjusted and unadjusted estimates.

**Figure 4:**
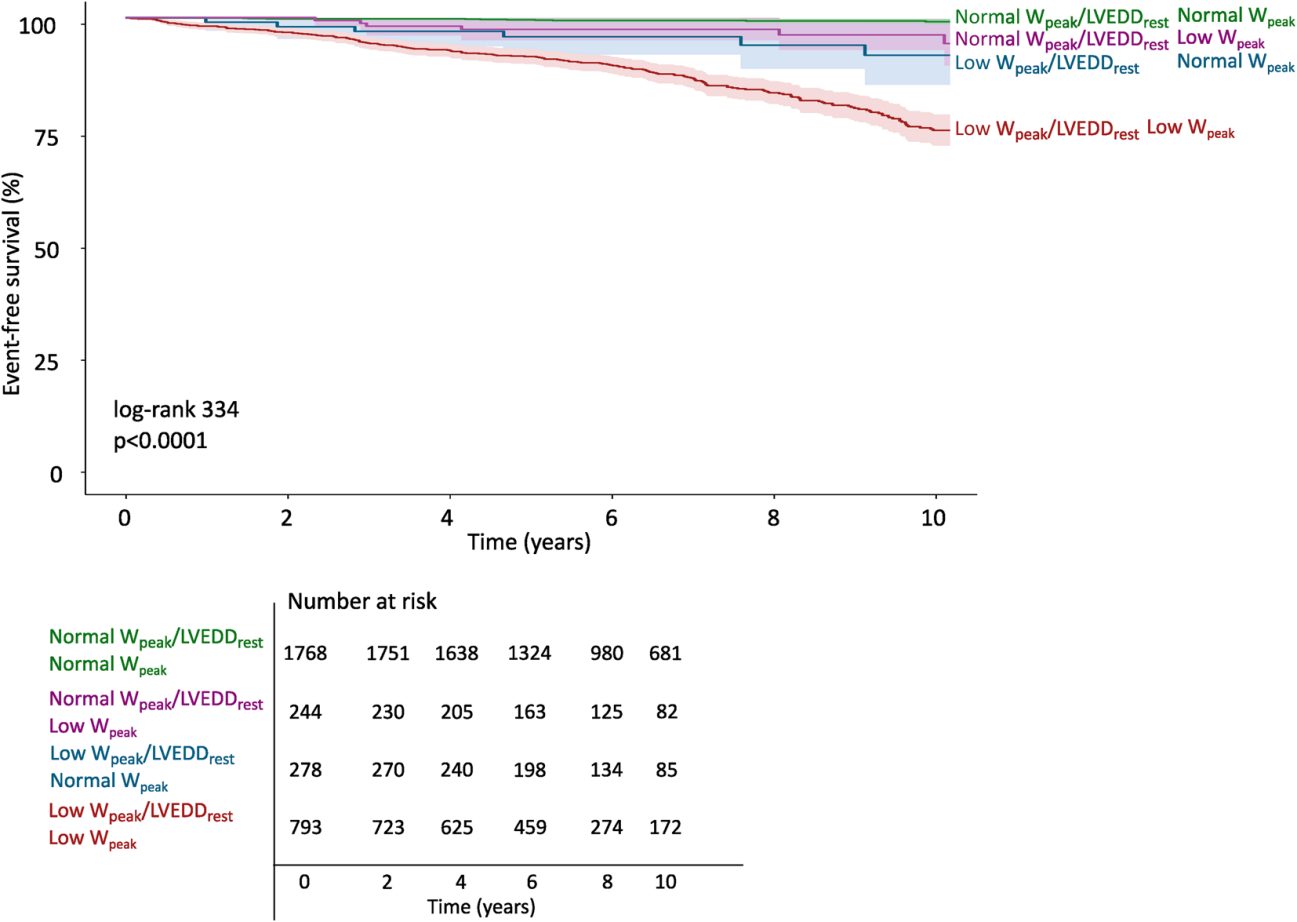
Time-to-event analysis for the combination normal/low W_peak_/LVEDD_rest_ and normal/low W_peak_ 3083 patients experiencing 249 events (cardiovascular death) over a median of 8.0 [IQR: 5.5, 11.1] years. A low W_peak_/LVEDD_rest_ as well as a low W_peak_ was defined as a value below the sex- and age-specific 5th percentile of lower-risk subjects.

The relative risk of CV mortality increased exponentially with lower W_peak_/LVEDD_rest_, as well as with lower W_peak_, Fig. 3. The combination of low W_peak_/LVEDD_rest_ and a low W_peak_ was associated with a particularly poor prognosis (adjusted HR 6.4 [4.0–10.3]) in reference to patients with normal W_peak_/LVEDD_rest_ and normal W_peak_), Fig. 4. In reference to those with a low W_peak_/LVEDD_rest_ but a normal W_peak_, the risk was three folded increased for those with both low W_peak_/LVEDD_rest_ and low W_peak_ (HR 3.1 [2.0–4.6]. Results for all-cause mortality are presented in Supplements (Table B, Figures A and B).

## Discussion

In a large consecutive cohort of patients undergoing clinical cycle ergometer exercise testing and an echocardiogram within 3 months, W_peak_/LVEDD_rest_ was a strong predictor of CV mortality, similar to W_peak_ in % of predicted, with identical prognostic strength when adjusted for age and sex.

We hypothesized that patients with a low exercise capacity and an enlarged LV would be at higher risk of cardiovascular death than patients with a low exercise capacity but a normally sized LV. While we did not find W_peak_/LVEDD_rest_ to provide incremental value to W_peak_ alone, we found that a low W_peak_/LVEDD_rest_ in combination with a low W_peak_ was associated with a particularly poor prognosis. A combination of a low W_peak_/LVEDD_rest_ and a low W_peak_ increased the risk of cardiovascular death by more than 600% (adjusted HR 6.4 [4.0–10.3])), in reference to those with normal W_peak_/LVEDD_rest_ and a low W_peak_. The risk of cardiovascular death increased exponentially with lower W_peak_/LVEDD_rest_. Therefore, it is possible that the W_peak_/LVEDD_rest_ could have a potential value in risk stratification of patients with heart failure in settings where more advanced markers for risk assessment are unavailable, e.g. cardiovascular magnetic resonance imaging or cardiopulmonary exercise stress testing with breathing-gas analysis^33,34^.

When describing the relation between exercise capacity and LV size, neither W_peak_ nor LVEDD were indexed to body size or age. When determining whether exercise capacity is reduced or not, W_peak_ should be related to anthropometric data (including sex and age)^13,18^. Reference values for exercise capacity using standardized cycle ergometer exercise test in Sweden has been published, in which age, sex and height, but not weight, are included in the regression equation^25^. In this study, we aimed to test the hypothesis that if exercise capacity was not increased in parallel to cardiac dimensions, the risk of cardiovascular death would increase. Since both exercise capacity and LVEDD are expected to be higher in larger individuals, this hypothesis does not require for any of the measures to be adjusted to body size. Previous studies have applied a similar strategy, i.e. assessing absolute values for peak VO2 and total heart volume or left ventricular dimension instead of indexing to body size^2,35^.

W_peak_ correlated with LVEDD_rest_ in the subgroup of lower-risk subjects, similar to previous reports in healthy subjects^1,3,4,36^. It has been shown previously that intensive endurance training leads to an increase in LV-volume in healthy subjects, as a cardiac morphological adaptation^4^, and Meyer et al. showed that patients with a low peak work rate tended to have smaller left ventricles. Our study showed no meaningful correlation between LV size and peak work rate in patients with HF or valvular heart disease. This is not unexpected since the underlying cause of LV remodeling in response to endurance training is different than, for example, after myocardial infarction or with valvular heart disease. In contrast, among lower risk subjects, the association between LV size and W_peak_ was stronger. In that way, an LV dilatation which is disproportionate to W_peak_ is a sign of LV disease, in our study represented by a low W_peak_/LVEDD_rest_^2^. Although this could not be elucidated in the present study, the potential role of W_peak_/LVEDD_rest_ in the discrimination of LV remodeling in response to exercise, i.e. athlete’s heart, and cardiac disease could warrant future studies.

### Limitations

Firstly, as we included patients with a clinical referral to exercise stress testing and echocardiography, there is a selection bias, limiting broad generalization of our findings. Secondly, LV volumes were not available, and may have provided better assessment of LV dilatation than LVEDD. It is also possible that including measures of both ventricles and atria may be a better way to index the peak work rate to cardiac size, since physiological cardiac adaptation generally affects all four cardiac chambers^2^.

Thirdly, echocardiographic data were obtained from clinical records and not by a standardized, study-specific protocol.

Since this is a retrospective analysis, we have no data on reproducibility of the LVEDD values.

## Conclusions

W_peak_/LVEDD_rest_ was associated with cardiovascular mortality but did not provide incremental prognostic value to W_peak_ alone. However, the combination of having a low W_peak_/LVEDD_rest_ and low W_peak_ was associated with a particularly poor prognosis.

## Supplementary Information

Supplementary Information.

## Data Availability

The data that support the findings of this study are available upon reasonable request to the corresponding author.
