# Supplementary material for: Prognostic value of peak work rate indexed by left ventricular diameter
Source: Sci Rep. 2023 May 31;13:8806. doi: 10.1038/s41598-023-35976-x (PMC10232525; doi:10.1038/s41598-023-35976-x)
Supplement: Supplementary file 1 — Supplementary Information. [file 41598_2023_35976_MOESM1_ESM.docx]

**Supplements**

**Supplemental Table A.** Definitions of diagnoses

| Diagnosis | Definition (either of) |
| --- | --- |
| Hypertension | 1. Prevalent diagnosis, hospital (outpatient, inpatient, admission; ICD10-codes) Hypertensive diseases (I10x-I15x) |
| Hyperlipidemia | Prevalent diagnosis, hospital (outpatient, inpatient, admission; ICD10-codes) Disorders of lipoprotein metabolism and other lipidemias (E78x) |
| Diabetes mellitus | 1. Prevalent diagnosis, hospital (outpatient, inpatient, admission; ICD10-codes) Type I (E10x), Type II (E12X), Other (E13x-E14x) |
| Heart failure | 1. Prevalent diagnosis, hospital (outpatient, inpatient, admission; ICD10-codes) Heart failure (I50x)  2. Prevalent diagnosis, hospital (outpatient, inpatient, admission; ICD10-codes) Cardiomyopathy (I42x-I43x)  3. Left ventricular ejection fraction <50% at the echocardiographic examination  4. Incident heart failure diagnosis within 3 months of the echocardiographic study (I50x) |
| Chronic obstructive pulmonary disease | 1. Prevalent diagnosis, hospital (outpatient, inpatient, admission; ICD10-codes) Bronchitis (J40x-J42x); Emphysema (J43); Other COPD (J44x) |
| Atrial fibrillation/flutter | 1. Prevalent diagnosis, hospital (outpatient, inpatient, admission; ICD10-codes) Atrial fibrillation and flutter (I48x) |
| Ischemic heart disease | 1. Prevalent diagnosis, hospital (outpatient, inpatient, admission; ICD10-codes) Angina pectoris (I20.9).  2. Prevalent diagnosis, hospital (outpatient, inpatient, admission; ICD10-codes) Acute myocardial infarction (I21x); Subsequent myocardial infarction (I22x); Complication following acute myocardial infarction (I23x); Other acute ischemic heart disease (I24x)  3. Prevalent diagnosis, hospital (outpatient, inpatient, admission; ICD10-codes) Unstable angina (I20.0) |
| Acute myocardial infarction | 1. Prevalent diagnosis, hospital (outpatient, inpatient, admission; ICD10-codes) Acute myocardial infarction (I21x); Subsequent myocardial infarction (I22x); Complication following acute myocardial infarction (I23x); Other acute ischemic heart disease (I24x) |
| Cerebrovascular disease | 1. Prevalent diagnosis, hospital (outpatient, inpatient, admission; ICD10-codes) (ICD>="I60") & (ICD<="I69") Subarachnoid haemorrhage (I60x); Intracerebral haemorrhage (I61x); Other nontraumatic intracranial haemorrhage (I62x); Cerebral infarction (I63x); Stroke, not 3 specified (I64x); Occlusion and stenosis of arteries, not resulting in cerebral infarction (I65x-166x); Other cerebrovascular diseases (I67x); Cerebrovascular disorders in diseases classified elsewhere (I68x); Sequelae of cerebrovascular disease (I69x). |
| Cardiovascular disease | 1. Prevalent diagnosis, hospital (outpatient, inpatient, admission; ICD10-codes)  ICD Ix |

**Supplemental Table B**. Associations between peak work rate to left-ventricular end-diastolic diameter at rest (W_peak_/LVEDD_rest_) and W_peak_ alone and LVEDD_rest_ and W_peak_ (% of predicted), respectively, and all-cause mortality (n=3,083 (592 events).

|  | **Unadjusted** | **Adjusted for  age and sex** | | **Final model*** |
| --- | --- | --- | --- | --- |
|  | HR [95%CI] | | HR [95%CI] | HR [95%CI] |
|  | C statistic  [95%CI] | | C statistic [95%CI] | C statistic [95%CI] |
| W_peak_/LVEDD_rest_ (W/mm) | 0.35 [0.31–0.39] | | 0.31 [0.27–0.35] | 0.38 [0.32 – 0.44] |
|  | 0.75 [0.73–0.77] | | 0.83 [0.81–0.84] | 0.84 [0.82–0.85] |
| W_peak_ (% of predicted) | 0.95 [0.95 - 0.96] | | 0.96 [0.95 - 0.96] | 0.97 [0.96-0.97] |
|  | 0.74 [0.72 - 0.76] | | 0.83 [0.81 - 0.84] | 0.89 [0.87 - 0.91] |
| LVEDD_rest_ (mm) | 1.04 [1.02–1.05] | | 1.05 [1.03–1.06] | 1.02 [1.00–1.04] |
|  | 0.54 [0.51–0.57] | | 0.77 [0.75–0.79] | 0.82 [0.80–0.83] |
| *Adjusted for age, sex, peak systolic blood pressure, ST depression, heart rate recovery, peak heart rate, left ventricular ejection fraction, E/e, heart failure, hypertension, myocardial infarction, diabetes mellitus, hyperlipidemia, and peripheral arterial disease.  Abbreviations: HR: hazard ratio; LVEDD: left ventricular end-diastolic diameter; W: Watt; W_peak_: peak work rate | | | | |

**
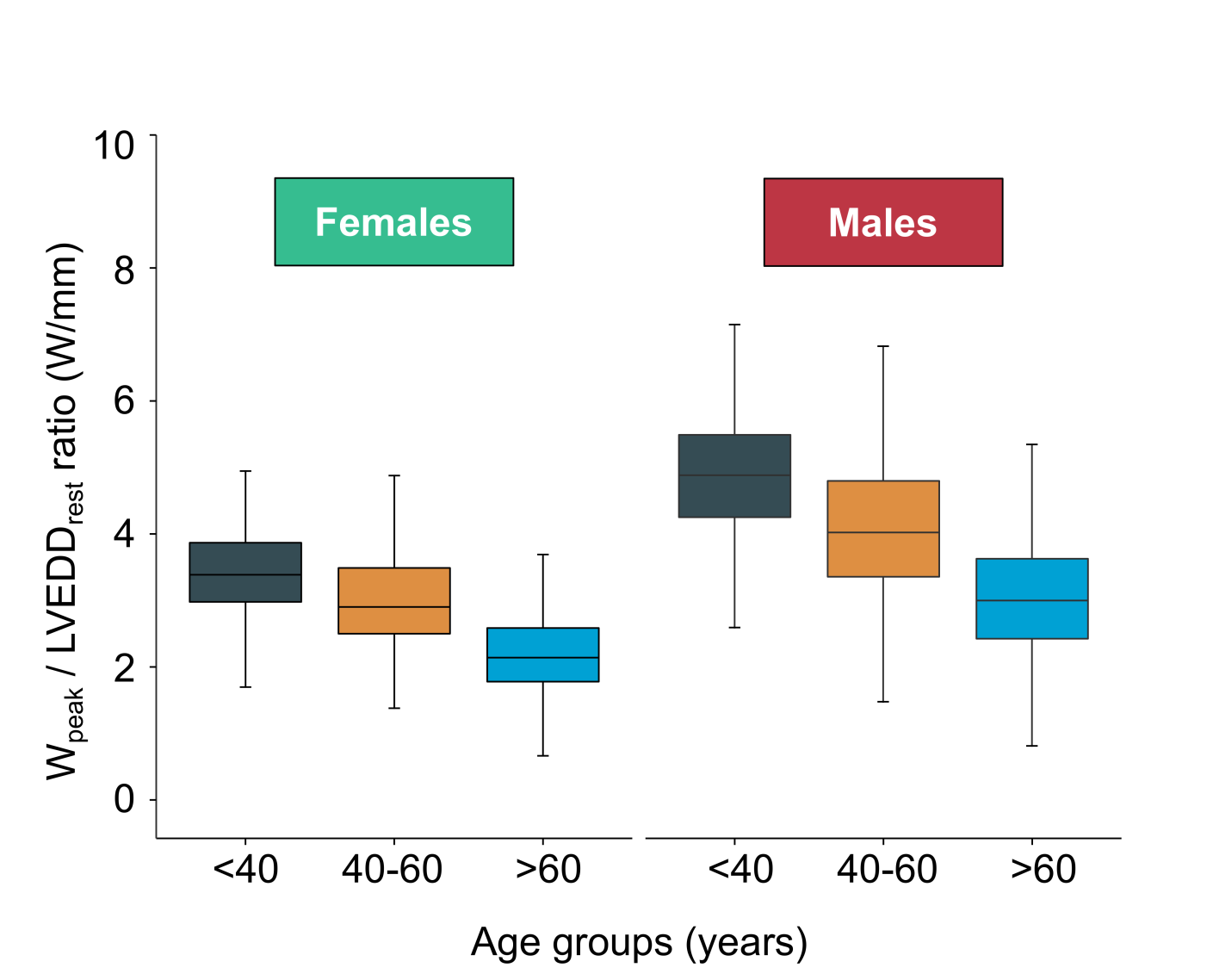
**

**Supplemental Figure A**. W_peak_/LVEDD_rest_ stratified by age and sex. W_peak_/LVEDD_rest_ is lower for females than for males, and decreases with age.

**
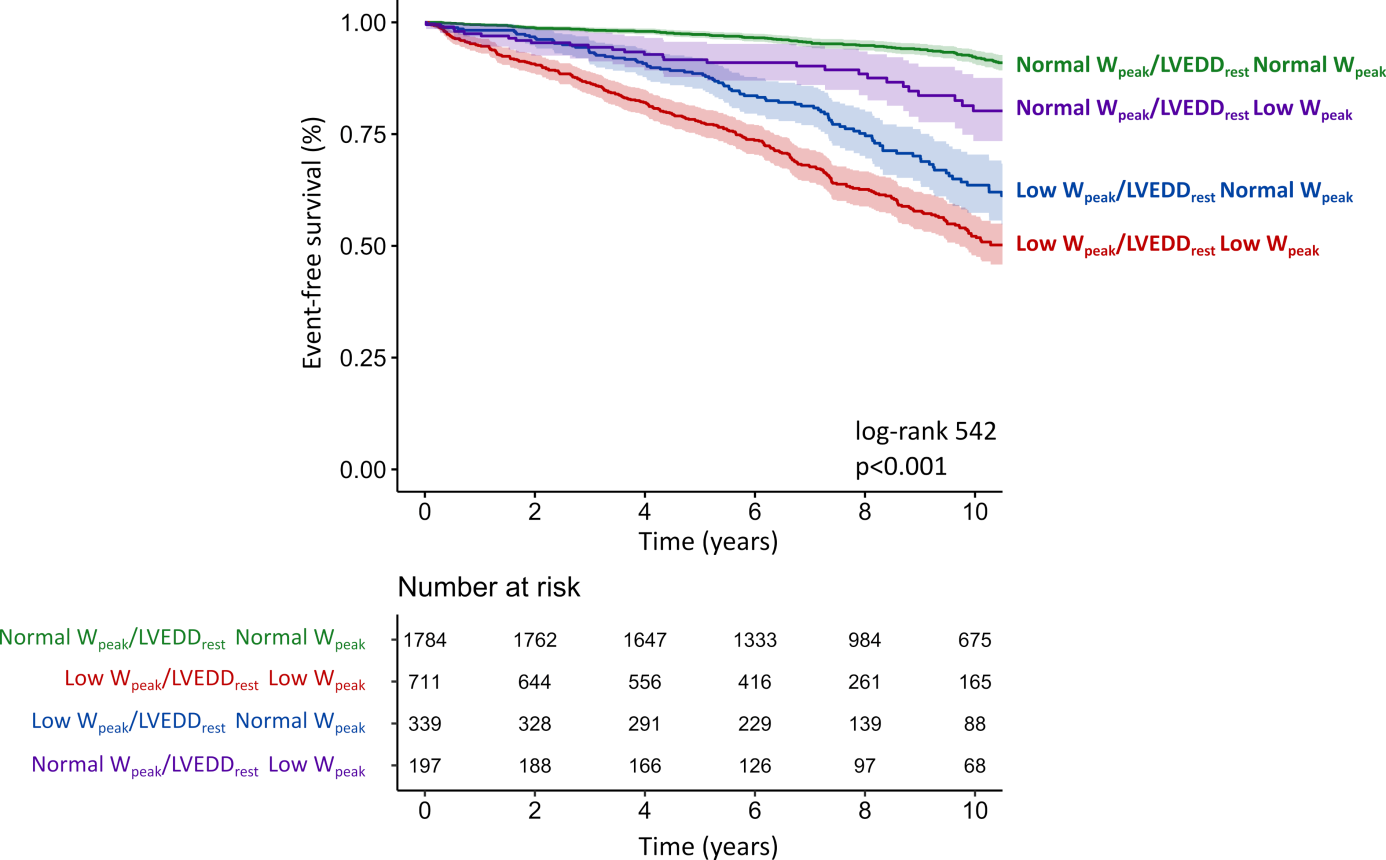
**

**Supplemental Figure B.** All-cause mortality Kaplan–Meier curve showing survival during a follow-up period over a median of 8.0 [IQR: 5.5, 11.1] years, based on combinations of normal/low W_peak_/LVEDD_rest_ and normal/low W_peak_. A low W_peak_/LVEDD_rest_ as well as a low W_peak_ was defined as a value below the sex- and age-specific 5^th^ percentile of lower-risk subjects.
